# Integrative Analysis of Normal Long Intergenic Non-Coding RNAs in Prostate Cancer

**DOI:** 10.1371/journal.pone.0122143

**Published:** 2015-05-01

**Authors:** Pushpinder Bawa, Sajna Zackaria, Mohit Verma, Saurabh Gupta, R Srivatsan, Bibha Chaudhary, Subhashini Srinivasan

**Affiliations:** 1 IBAB, Institute of Bioinformatics and Applied Biotechnology, Bangalore, Karnataka, India; 2 Manipal University, Manipal, Karnataka, India; Harbin Medical University, CHINA

## Abstract

Recently, large numbers of normal human tissues have been profiled for non-coding RNAs and more than fourteen thousand long intergenic non-coding RNAs (lincRNAs) are found expressed in normal human tissues. The functional roles of these normal lincRNAs (nlincRNAs) in the regulation of protein coding genes in normal and disease biology are yet to be established. Here, we have profiled two RNA-seq datasets including cancer and matched non-neoplastic tissues from 12 individuals from diverse demography for both coding genes and nlincRNAs. We find 130 nlincRNAs significantly regulated in cancer, with 127 regulated in the same direction in the two datasets. Interestingly, according to Illumina Body Map, significant numbers of these nlincRNAs display baseline null expression in normal prostate tissues but are specific to other tissues such as thyroid, kidney, liver and testis. A number of the regulated nlincRNAs share loci with coding genes, which are either co-regulated or oppositely regulated in all cancer samples studied here. For example, in all cancer samples i) the nlincRNA, TCONS_00029157, and a neighboring tumor suppressor factor, SIK1, are both down regulated; ii) several thyroid-specific nlincRNAs in the neighborhood of the thyroid-specific gene TPO, are both up-regulated; and iii) the TCONS_00010581, an isoform of HEIH, is down-regulated while the neighboring EZH2 gene is up-regulated in cancer. Several nlincRNAs from a prostate cancer associated chromosomal locus, 8q24, are up-regulated in cancer along with other known prostate cancer associated genes including PCAT-1, PVT1, and PCAT-92. We observe that there is significant bias towards up-regulation of nlincRNAs with as high as 118 out of 127 up-regulated in cancer, even though regulation of coding genes is skewed towards down-regulation. Considering that all reported cancer associated lincRNAs (clincRNAs) are biased towards up-regulation, we conclude that this bias may be functionally relevant.

## Introduction

The promise of the Human Genome Project was to deliver hundreds of thousands of proteins for use as drug targets. However, to everyone’s surprise, large-scale annotation efforts by large consortia, such as ENCODE project[1], delivered tens of thousands of drug targets of a different kind; non-coding RNAs. It is now known that majority of the human genome is transcribed even though only a small fraction translates into proteins. It is now understood that a large number of non-coding RNAs, both long and short, play critical roles in the complex regulation of the relatively small number of coding proteins that are essential for life.

Of the diverse types of non-coding RNAs, long intergenic non-coding RNAs (lincRNA) are attractive because they can be easily discovered with high confidence from existing RNA-seq datasets and correlated with gene expression information from the same dataset using existing bioinformatics tools. More recently, tens of thousands of lincRNAs have been discovered from RNA-seq datasets from diverse normal human tissues, here to referred nlincRNAs, such as the Illumina Body Map[2]. The functional roles of these nlincRNAs are yet to be established.

Despite the fact that lincRNAs are new to cancer biology and their molecular mechanisms still in its infancy, several review papers have already appeared in the literature detailing progress in this area to date[3] [4]. Of the roughly 60+ lincRNAs that have been shown to be associated with various cancer types, majority of them are up-regulated in cancer[3] [4] and only a few lincRNAs are shown to be down-regulated in cancer samples including GAS5[5] and MEG3[6].

Recent reports linking expression levels of lincRNA with cancer offer an excellent opportunity for establishing functional role of lincRNAs in regulating gene expression. One of the most exhaustive search for lincRNAs associated with prostate cancer include, identification of 121 lincRNAs, called PCATs (Prostate Cancer Associated Transcripts) discovered from 102 disease stratified prostate tissues and cell lines[7]. Out of these, PCAT-1 inhibition with siRNA is shown to reduce proliferation of celllines expressing high-levels of PCAT-1. Since publication of this report, PCAT-1 over-expression has been shown to be a biomarker in colorectal cancer[8]. More recently, lincRNAs from RNA-seq data from a large number of lung cancer samples from the public repository has been used to identify 111 lung cancer associated lincRNAs, called LCALs[9]. The bias of lincRNAs towards up-regulation in cancer requires interrogation.

It is tempting to conclude that the bias towards up-regulation of lincRNAs in cancer, in the large-scale efforts cited above, may results from the practice of discovering lincRNAs from cancer samples. Here, our aim was to perform an integrative analysis of both coding and non-coding nlincRNAs (lincRNAs discovered from normal human tissues) across multiple RNA-seq datasets pertaining to prostate cancer from public repository to both address this bias and discover novel co-regulation of genes and nlincRNAs.

## Materials and Methods

### Datasets used

We have used two RNA-seq datasets from NCBI public repository generated by two independent groups with accession IDs of SRP002628 and ERP000550. As shown in Table 1, 5 tumor-normal pairs from SRP002628 and 7 from ERP000550 datasets are considered in this study either because the corresponding pairs were not available for some individuals or the depth of sequencing were not compatible to obtain good statistics. These two datasets are from two diverse demographics. For example, patients selected to generate data within the accession ERP000550 are Chinese in origin and, although the demography of patients in the SRP002628 dataset is not known, it is safe to assume that the individuals considered to generate data within the accession SRP002628 are not Chinese. Table 1 gives the depth, individual accession IDs and mapping percentages for each sample in these datasets.

**Table 1:** RNA-seq runs selected from SRP002628 and ERP000550 to obtain the signature and those from ERP00550 used in the validation.

ERP000550					
Normal			Tumor		
Accession	Number of reads	Percent Mapped	Accession	Number of reads	PercentMapped
ERR031029_N02	35534313	74.33	ERR031030_C02	32289266	77.63
ERR031031_N03	31921622	70.33	ERR031032_C03	32319406	73.02
ERR031039_N07	38401723	74.07	ERR031040_C07	33974921	77.36
ERR031043_N09	34266043	75.15	ERR031044_C09	34758125	75.69
ERR031017_N10	34536162	83.17	ERR031018_C10	34007787	82.16
ERR031023_N13	31245264	77.53	ERR031024_C13	37576110	79.35
ERR031025_N14	33918112	70.52	ERR031026_C14	36886097	73.57
Validation Dataset					
ERR031033_N04	33965736	72.88	ERR299297_C04	34505542	74.25
ERR299299_N06	36320661	72.60	ERR031038_C06	35679519	77.13
ERR031041_N08	33191569	76.51	ERR031042_N08	34988865	77.83
ERR031019_N11	36250477	79.04	ERR299295_C11	34718521	80.04
ERR299296_N12	32272887	70.55	ERR031022_C12	36820858	72.47
SRP002628					
**Normal**			**Tumor**		
Accession	Number of reads	PercentMapped	Accession	Number of reads	PercentMapped
SRR057658_N23	14676269	73.11	SRR057642_C23	15212560	72.26
SRR057657_N19	11914701	68.06	SRR057641_C19	16307495	71.15
SRR057656_N15	14236982	70.08	SRR057638_C15	16274538	72.38
SRR057655_N13	14747638	69.06	SRR057637_C13	15530810	72.03
SRR057658_N11	14761953	67.62	SRR057636_C11	10996701	59.43

For profiling known and novel lincRNAs we used GENCODE (http://www.gencodegenes.org/) and lincRNA-catalog (http://www.broadinstitute.org/genome_bio/human_lincrnas/?q=lincRNA_catalog). Also for gene expression analysis we have used the table browser from the URL https://genome.ucsc.edu/cgi-bin/hgTables for hg19 with track as refseq genes and output format as BED.

For baseline expression of coding gene data we have used both E-MTAB-513 with 16 and E-MTAB-1733 with 27 normal human tissues from Expression Atlas under ArrayExpress. In this study we have used a FPKM value of less than 0.5 to make baseline null calls and FPKM value of greater than 100 to call them tissue-specific.

### Method used to compute transcript expression

Selected datasets were mapped to hg19 reference genome using Bowtie[10] with percentage mapped shown in Table 1. For the reads under the accession SRP002628 the entire length of the reads, which is 36mer, were mapped. However for reads under ERP000550 25 bases were trimmed from both ends of the reads of length 90 leading to mapping of 40mers from the middle. The tool coverageBed from BEDTools were used to extract count per transcript per sample using the annotation files and lincRNA-catalog mentioned in the above section. These individual count files were collated into a table with rows representing transcripts.

### Computing differential expression

For computing differential expression we selected two widely used count-based R packages, edgeR[11] and DESeq[12]. Although these two methods are very similar they differ in the use of dispersion. The package edgeR uses single common dispersion factor as opposed to a flexible variance estimation used by the package DESeq. The important distinction of edgeR is that it is anti-conservative to low expressed genes and more conservative to highly expressed genes. Where as, the flexible dispersion model used by DESeq allows for lesser bias in selection of genes based on their expression levels. This way DESeq and edgeR complement each other in the selection of differentially expressed genes. In other words edgeR is more sensitive to outliers where as DESeq is less sensitive to outliers but provides unbiased outcome through the dynamic range[13].

DESeq and edgeR both accepts a collated count file as input and produce single p-value and log fold-change per transcript per dataset representing the overall differential expression state of the transcripts in the annotation file between two given states, tumor and matched non-neoplastic tissues. To obtain cancer-specific transcripts that are statistically significant we used a p-value cutoff of less than 0.05 and a abs (log fold change to base 2) greater than 1.0 for coding genes and greater than 0.59 for nlincRNAs. The variation in the filtering criteria chosen for fold change is to reflect the relatively lower levels of nlincRNA expression compared to coding genes reported in the literature [2].

### Clustering heatmap

For generating heatmaps we used Pearson Correlation Coefficient and for dendrograms we used Euclidian distance using ‘pheatmap’ and ‘hclust’ functions from R statistical package respectively. In order to produce heatmaps for samples across datasets additional normalization was required to account for the variation in the dispersion in gene expression levels between datasets stemming from different sample preparation protocols used by different investigators. Although RPKM values are computed to normalize expression levels across samples, this normalization is sufficient to account for variation in sample preparation protocol. Normalizing by rows, representing transcripts, between datasets by dividing them by row average was used to handle the differential dispersion between the two datasets stemming from variation in sample preparation protocols. Such an approach has already been implemented in DESeq package for samples within a given dataset[12].

### Validation of datasets

To show that the two selected datasets are suitable for profiling non-coding RNAs, the expression levels of known lincRNAs, which are reported as implicated in cancer, have been profiled across the two RNA-Seq datasets selected for this study. We have identified that several prostate-cancer associated lincRNAs are up-regulated in a tumor-specific fashion in both these datasets. For example, PCAT-1, a prostate cancer associated lincRNA from the gene-desert locus in chromosome 8q24 is significantly up-regulated in all tumor samples from both datasets compared to adjacent non-neoplastic tissues. Several other prostate and other cancer-specific lincRNAs, such as PVT1, PCA3, CCAT-1 PCAT-92, PCAT-114, PCAT-120, PCAT-19, PCAT-27, PCAT38, PCAT-39, PCAT-43, PCAT-59, PCAT-72, PCAT-80, and PCAT-83 are also found to be up-regulated in both datasets in a cancer-specific fashion. These findings, not only authenticates the use of these two datasets for nlincRNA profiling but provide additional validation for these newly minted lincRNAs in prostate cancer.

### Gene Network

Gene network in this manuscript is created by GeneMANIA, a package under Cytoscape [14].

## Results and Discussion

### Profiling Coding Genes

**Fig 1:**
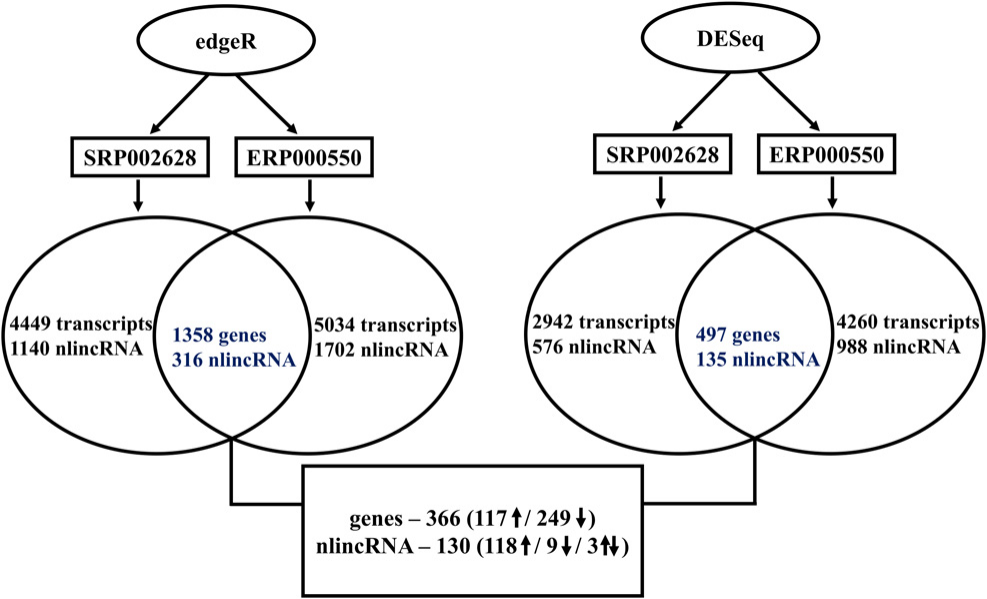
Flowchart with converging significance of genes and nlincRNAs differentially regulated from the two datasets and the two methods.

Gene expression profiling of the two RNA-seq datasets, SRP002628 [15] and ERP000550 [16], were performed using two commonly used R statistical analysis packages, edgeR [11] and DESeq [12]. Genes with p-values of less than 0.05 and absolute log fold changes (base 2) greater than 1.0 are used to make a call that a gene is regulated in prostate cancer. Converging numbers of significantly regulated genes at each stage of the analysis pipeline is presented in Fig 1. Using edgeR, 4449 and 5034 transcripts are found differentially regulated from the two datasets, SRP002628 and ERP000550 respectively. Out of these, 1358 transcripts representing 786 genes are found commonly regulated between the two datasets with 302 genes up-regulated and 455 genes down-regulated in cancer. Interestingly, only 29 genes showed opposite expression pattern in the two datasets. Similarly, using DESeq package 2942 and 4260 transcripts are identified as differentially regulated in prostate cancer from the two datasets, SRP002628 and ERP000550 respectively. The 881 common transcripts represent 497 genes, which are regulated in prostate cancer with 180 genes up- and 313 genes down-regulated. Again, only 4 genes display opposite regulation in the two datasets based on DESeq pipeline.

**Fig 2:**
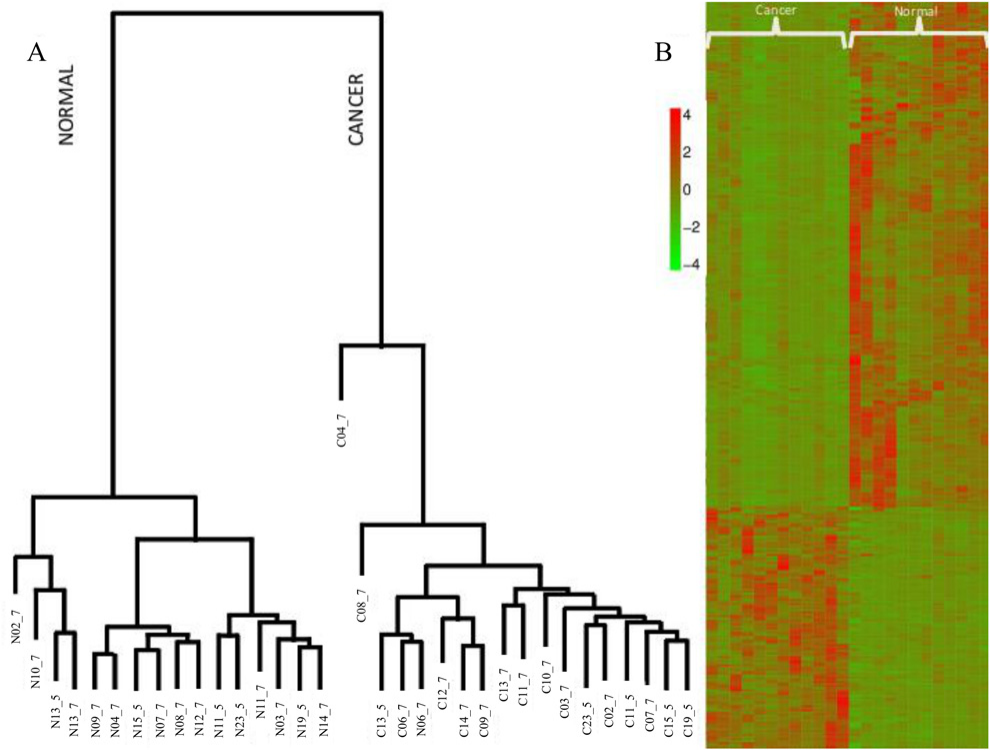
A) dendrogram and B) Heatmap of the 366 genes differentially regulated in cancer sample in both datasets.

The list of differentially expressed genes (DEGs) from the two datasets using the two methods, edgeR and DESeq, is listed in S1 Table, which contain 366 coding genes with 117 up- and 249 down-regulated in prostate cancer. Interestingly, with the exception of only one gene, all are regulated in the same direction in both cancer datasets (Fig 2). In Fig 2, it is also shown that the row-wise normalized RPKM values from the two datasets for all the 366 DEGs, clusters all the 12 cancer and 12 normal samples in two distinct clades. Also shown in Fig 2, are the clustering of five additional samples from the accession ERR000550, not used in extracting the signature, in the respective clades.

**Fig 3:**
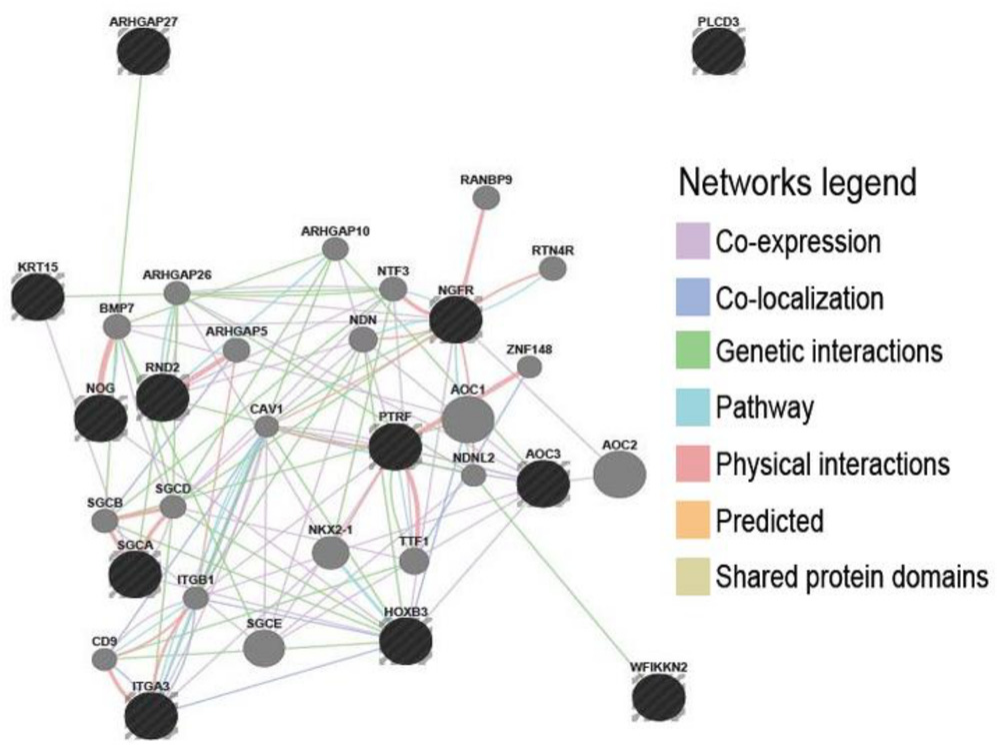
Interaction network of inactivated gene cluster from 17q21 locus using GeneMania.

Gene enrichment studies on the 366 genes suggests inactivation of genes in both 17q21 and 19q13 loci, which are both reported as prostate cancer susceptibility loci [17], [18], [19]. Fig 3 shows the gene network for loci 17q21 and 19q13. Interestingly, the genes inactivated in 17q21, including KRT15, ITGA, AOC3, HOXB3, RND2, SGCA, WFKKN2, ARHGAP27, NGF, and NOG, are implicated in cell-cell interaction, cytoskeletal reorganization, extra-cellular matrix and cell death; lack of which could cajole epithelial to mesenchymal transformation and migration.

### Profiling nlincRNAs

A total of fourteen-thousand three hundred and fifty-three (14,353) lincRNAs, referred here to as nlincRNAs, has been reported to be expressed in various normal human tissues[2]. Out of these, there are 9,600 nlincRNAs that show very low evidence of transcription in prostate normal and as low as 196 nlincRNAs are reported as specific to prostate tissues.

As shown in Fig 1, using edgeR, 1140 (773 up and 367 down-regulated) and 1702 (1258 up- and 444 down-regulated) nlincRNAs are found differentially regulated from the two datasets, SRP002628 and ERP000550, respectively. Out of these, 316 nlincRNAs (252 up and 42 down) are commonly regulated in prostate cancer in both datasets. Similar analysis using DESeq pipeline resulted in 576 (383 up and 193 down) and 988 (867 up and 121 down) nlincRNAs regulated in prostate cancer in both datasets, SRP002628 and ERP000550, respectively. The number of commonly differentially regulated nlincRNAs in both datasets using DESeq package is 135 with 124 up- and 9 down-regulated in cancer.

**Fig 4:**
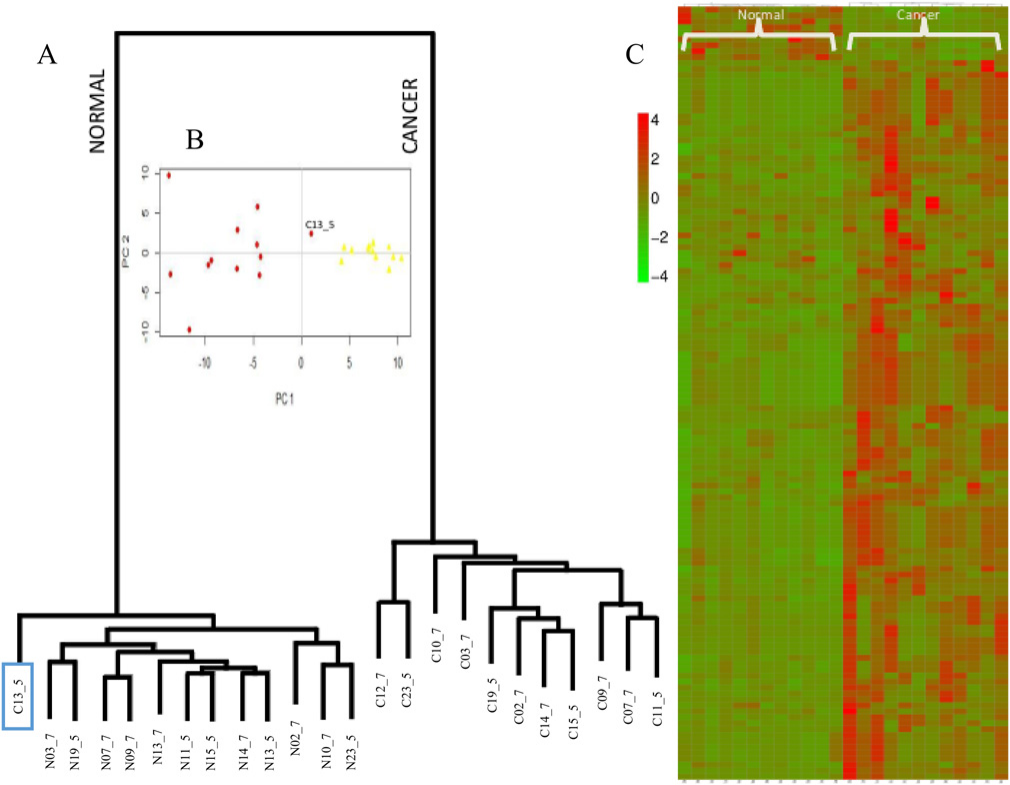
A) dendrogram, B) principal component and C) Heatmap of the 130 differentially regulated lincRNAs in all cancer samples from both datasets using both edgeR and DESeq.

The number of nlincRNAs that are found differentially regulated in cancer in both datasets using both edgeR and DESeq methods, is 130 with 118 up-, 9 down- and as low as 3 oppositely regulated. Fig 4 shows the heatmap of the 127 nlincRNAs, listed in S2 Table, that are differentially regulated in cancer. With the exception of C13_5, one of the cancer sample from accession SRP002628, the 127 nlincRNAs allow clustering of all the cancer and normal samples in the respective clades. Using principal component analysis, shown in Fig 4, it is confirmed that C13_5 is more normal-like.

Out of the 127 differentially regulated nlincRNAs, 58 have null baseline expression in prostate normal tissue according to both in-house efforts and the report by Broad Institute [2]. Of these nlincRNAs, many are testis-specific and a number of them are thyroid-specific. As shown in the S2 Table, profiling of these nlincRNAs in prostate cell-lines from RNA-seq dataset with accession IDs of SRP004637[7], reveal that 12 out of the 58 display significant expression in one or more of the three prostate celllines. This trend is observed in prostate cancer associated coding genes such as EZH2[20], which is differentially up-regulated in cancer samples from both datasets display baseline null expression in prostate. Also, as shown in S3 Table, many reported prostate cancer associated lincRNAs, like PCAT-1, PCA3, PCAT-92, PCAT-114, PCAT-120-PCAT-27, PCAT-38, PCAT43, PCAT72, and PCAT-80 [7], which are also differentially up-regulated in cancer in both datasets, has no overlapping nlincRNAs according UCSC tracks.

There are many nlincRNAs from chromosome 8q24 locus, listed in Table 2, that are expressed in normal human tissues. While a number of nlincRNAs share exons with known lincRNAs, such as PVT1 and CCAT1, several others including TCONS_00014535 (BC042052, CASC11), TCONS_00015171 (BC106081), TCONS_00015167 (PCAT2), TCONS_00015170 and TCONS_00015168 (JX003871), TCONS_00015498, TCONS_00015165 and TCONS_00015166 are novel nlincRNAs that are differentially up-regulated in prostate cancer in at least one of the two datasets used in this study. Again, many of these are specific to testis and liver and are not expressed in normal prostate.

**Table 2:** Lists all the nlincRNAs within the 8q24 loci p-value and fold-change in the two datasets by the two methods.

Locus	Tissue Specificity	TCONS_ID	Chinese				5 Sample				Mapping to
			edgeR		DESeq		edgeR		DESeq		
			p-value	FC	p-value	FC	p-value	FC	p-value	FC	
PCAT-1 /CCAT-1	-	TCONS_00015165	0.07	1.08	0.08	1.30	0.73	-0.13	0.83	-0.19	-
	Testes	TCONS_00015166	0.01	1.65	0.00	1.83	0.06	0.51	0.25	0.46	-
	Testes	TCONS_00015167	0.06	1.22	0.09	1.44	0.43	0.69	0.62	0.72	PCAT2
	-	TCONS_00015168	0.00	2.17	0.00	2.36	0.03	0.67	0.15	0.64	JX003871
		TCONS_00015170	0.00	2.16	0.00	2.34	1.00	0.20	0.98	0.19	
		TCONS_00015498	0.00	1.85	0.00	2.03	0.16	0.55	0.24	0.49	-
	Liver	TCONS_00015169	0.00	2.08	0.00	2.24	0.00	1.34	0.00	1.29	CCAT1
		TCONS_00015171	0.00	1.83	0.00	2.00	0.00	1.92	0.01	1.90	BC106081
		TCONS_00014531	0.01	1.77	0.03	1.90	0.00	1.80	0.01	1.85	CCAT1
PVT-1 /MYC	-	TCONS_00015353	0.09	1.11	0.04	1.28	0.25	0.32	0.44	0.28	PVT1
		TCONS_00015354	0.09	1.11	0.04	1.28	0.05	0.55	0.20	0.50	
	-	TCONS_00015355	0.02	1.42	0.01	1.60	0.00	1.05	0.01	1.01	
		TCONS_00015356	0.02	1.41	0.01	1.59	0.22	0.52	0.21	0.46	
		TCONS_00015357	0.03	1.27	0.02	1.43	0.00	1.34	0.00	1.33	
		TCONS_00015358	0.01	1.44	0.01	1.62	0.05	0.96	0.02	0.93	
	-	TCONS_00014535	0.01	1.65	0.01	1.79	0.82	0.09	0.78	0.05	CASC11

### Co-regulation of lincRNAs and neighboring genes

**Fig 5:**
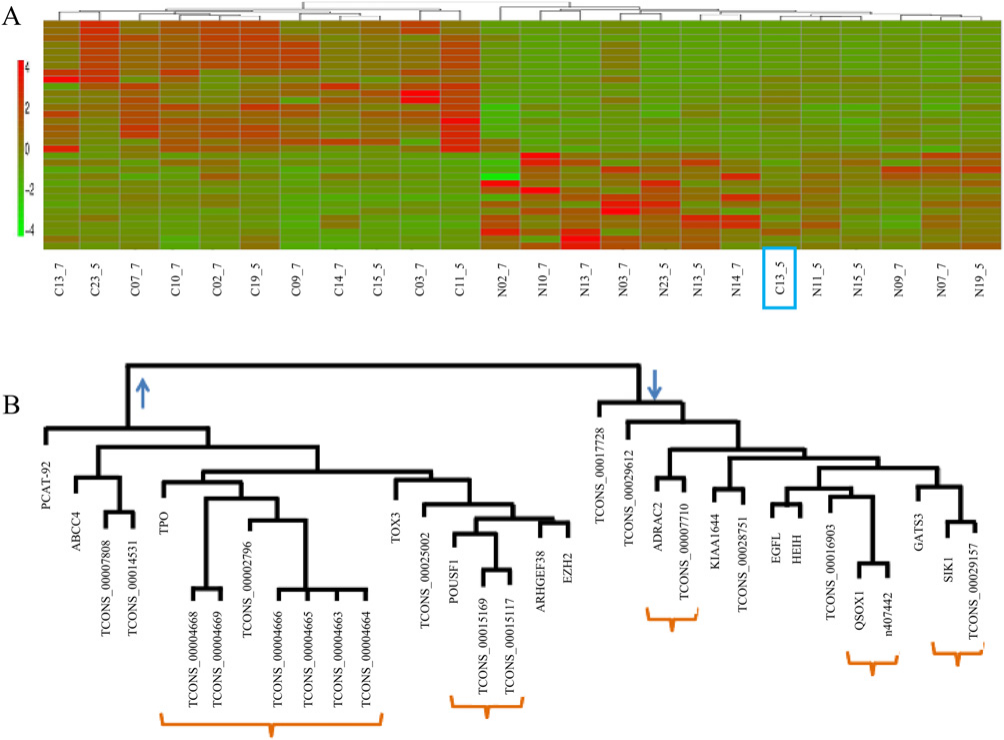
A) Heatmap and B) dendrogram for all the lincRNAs along with their neighboring genes, which are also differentially regulated. Red arrows indicate clusters of neighboring gene and lincRNAs.

As shown in Fig 5, 15 differentially regulated nlincRNAs out of the 127 are near 12 differentially regulated genes on various chromosomes. Table 3 provide significance of nlincRNAs and their respective coding gene. For example, the nlincRNA, TCONS_00029157, and a known tumor suppressor factor, SIK1, are both down regulated in all cancer samples. Reduced SIK1 expression is correlated with poor prognosis in two large human breast cancer data sets and is linked with p53-dependent anoikis that may be targeted during tumerogenesis [21].

**Table 3:** Lists p-value and fold-change for all the nlincRNAs that are differentially regulated along with their neighboring genes in cancer.

Gene / TCONS_ID	DESeq				EdgeR			
	5 Sample		Chinese Sample		5 Sample		Chinese Sample	
	pvalue	foldchange	pvalue	foldchange	pvalue	foldchange	pvalue	foldchange
TCONS_00029157	0.02	-1.23	0.00	-2.02	0.01	-1.17	0.00	-2.20
SIK1	0.00	-0.96	0.01	-1.50	0.00	-1.02	0.04	-1.51
TCONS_00004663	0.00	2.54	0.00	2.69	0.00	2.52	0.00	2.53
TCONS_00004664	0.00	3.01	0.00	2.69	0.00	3.00	0.00	2.53
TCONS_00004665	0.00	3.25	0.00	2.69	0.00	3.27	0.00	2.53
TCONS_00004666	0.00	2.66	0.00	2.69	0.00	2.66	0.00	2.53
TCONS_00004668	0.00	1.73	0.00	3.20	0.00	1.75	0.00	2.93
TCONS_00004669	0.00	1.99	0.00	3.20	0.00	1.99	0.00	2.93
TPO	0.00	2.37	0.00	3.56	0.00	2.31	0.00	3.56
TCONS_00010581	0.01	-0.88	0.00	-0.61	0.01	-0.96	0.00	-0.73
EZH2	0.01	1.15	0.03	1.26	0.01	1.10	0.00	1.24
TCONS_00025002	0.04	0.87	0.05	1.31	0.01	0.91	0.02	1.13
TOX3	0.00	1.91	0.01	1.40	0.01	1.86	0.01	1.40
TCONS_00017728	0.04	-1.85	0.00	-2.80	0.01	-1.81	0.00	-3.00
GATA3	0.05	-0.86	0.00	-2.32	0.06	-0.92	0.00	-2.33
TCONS_00010086	0.03	1.07	0.01	2.04	0.01	1.13	0.00	1.83
ADAMTS19	0.45	0.62	0.00	4.90	0.65	0.56	0.17	4.88
TCONS_00014531	0.01	1.85	0.03	1.90	0.00	1.80	0.01	1.77
TCONS_00015169	0.00	1.29	0.00	2.24	0.00	1.34	0.00	2.08
TCONS_00015171	0.01	1.90	0.00	2.00	0.00	1.92	0.00	1.83
POU5F1B	0.01	1.60	0.01	1.42	0.11	1.62	0.00	1.41
TCONS_00028940	0.00	3.10	0.02	3.08	0.00	3.09	0.00	3.02
TMPRSS2	0.82	-0.09	0.30	0.54	0.88	-0.15	0.32	0.54

The thyroid-specific TPO gene is up-regulated in prostate cancer along with a few thyroid-specific nlincRNAs, TCONS_00004663–4666 and TCONS_00004668–4669. TPO is one of the genes known to be associated with oxidative stress. It has been shown that lens epithelial derived growth factor p75 (LEDGF) in PC3, results in the change in TPO expression[22]. This change is likely to play a protective role against oxidative stress and chemotherapeutic drugs.

TCONS_00010581, an isoform of HEIH, which is known to be up-regulated in hepatocellular carcinoma [23], is in the proximity of the gene EZH2 of the polycomb complex-2 [24] The gene EZH2 is also found up-regulated in all cancer samples compared to adjacent non-neoplastic tissues in both datasets.

A testis-specific nlincRNA, TCON_00025002, is in the neighborhood of the gene TOX3 on chromosome 16, which are both up-regulated in a cancer-specific fashion in our study. TOX3 is a high motility group box protein involved in mediating calcium-dependent transcription. TOX3 maps to the known triple-negative breast cancer susceptibility locus; a mutation in this locus in implicated in the development of breast cancer[25]. A SNP in TOX3 gene is also implicated in pancreatic[26] and lung cancer[27].

Prostate-specific nlincRNAs, TCONS_00017728 and TCONS_00010086, are found to be in the vicinity of GATA3 and ADAMTS19 genes respectively. All four, including the nlincRNAs and genes, are down regulated in a cancer-specific fashion in this study. GATA3 is an important transcription factor known to be involved in androgen regulation of PSA gene[28]. A global methylation pattern in androgen sensitive and androgen independent prostate cancer shows a significant difference in the methylation pattern in GATA3 under these two conditions[29]. Tumor biopsies and various cancer cell lines have show high levels of expression of ADAMTS19 in osteosarcomas[30].

A few liver-specific nlincRNAs, TCONS_00014531, TCONS_00015169 and TCONS_000015171, are all up-regulated in prostate cancer in this study along with the neighboring pseudogene POU5F1, which is adjacent to MYC locus in the major prostate cancer susceptibility locus in 8q24[31]. Another liver specific TCONS_00016903 is juxtaposed to gene EGFL7, both recorded as down-regulated in our analysis. Contrary to our findings EGFL7 has been shown to have an elevated expression in various cancer types including lung cancer, breast cancer, prostate cancer and hepatocellular carcinoma[32]. However, there has been a report of a microRNA, miR-126, located within the intron of EGFL7, which is shown to be down-regulated in cancer cell lines and in primary bladder and prostate tumors[33].

Among the more interesting nlincRNAs, TCONS_00028940, in the neighborhood of the gene TMPRSS2, is highly differentially expressed in all cancer samples studied here. The TMPRSS2-ERG gene fusion is one of the most widely spread chromosomal rearrangements in carcinomas [34], although the gene TMPRSS2 is not expressed in a cancer-specific fashion in samples studied here We find that this nlincRNA shows significant expression in VCaP and not in PC3 and LnCaP.

## Conclusion

Recently, more than fourteen thousand lincRNAs have been discovered from large number of normal human tissues, suggesting that these normal lincRNAs (nlincRNAs) play a role in normal biology. It can be hypothesized that nlincRNAs with gene regulatory functions in normal conditions may actually be down-regulated in cancer. For this purpose, here we have attempted to take two independently generated RNA-seq datasets from demographically diverse cohort to profile both protein coding genes and nlincRNAs. We have identified 127 nlincRNAs that are not only significantly regulated in cancer samples from both datasets but could be used to cluster data from samples, not used in this study, by disease context. Contrary to our hypothesis, profiling of coding genes and nlincRNAs suggests that a majority of the nlincRNAs are up-regulated in cancer even though 2 fold more protein coding genes are down-regulated in cancer. This together with the activation of many non-coding genes in 8q24 and inactivation of many coding genes in 17q21 and 19q13 loci would suggest systems level activation of many nlincRNAs during cancer.

We have found that a number of coding genes and nlincRNAs specific to other tissues with baseline null expression in prostate tissue are up-regulated in prostate cancer. Perhaps these genes and nlincRNAs are responsible for the loss of cellular identity leading to tumerogenesis. To our knowledge this is the first attempt to profile nlincRNAs along with coding genes in cancer. We believe that the approach used here for functional characterization of nlincRNAs will allow researchers to advance the understanding of the role of nlincRNAs in normal and disease biology, in general.

## Supporting Information

S1 Table Genes differentially regulated in cancer samples from both datasets identified using both DESeq and edgeR analysis pipelines. Columns 2–5 gives the average p-values and fold change obtained from DESeq and edgeR pipeline for both SRP002628 and ERP000550.
(XLS)

S2 Table Lists p-value and fold-change for all the nlincRNAs differentially regulated in both datasets using both methods. The table lists neighboring genes along with their respective p-value and fold-change in the two datasets. Last three columns lists the RPKM values for these lincRNAs in three prostate celllines.
(XLS)

S3 Table Lists p-value and log fold-change for known lincRNAs that are differentially regulated in both datasets along with the status of the transcript overlapping with nlincRNAs on UCSC browser. (XLS)
